# Hypoxia Enhances the Antiglioma Cytotoxicity of B10, a Glycosylated Derivative of Betulinic Acid

**DOI:** 10.1371/journal.pone.0094921

**Published:** 2014-04-17

**Authors:** Sebastian Fischer, Michael W. Ronellenfitsch, Anna-Luisa Thiepold, Patrick N. Harter, Sebastian Reichert, Donat Kögel, Reinhard Paschke, Michel Mittelbronn, Michael Weller, Joachim P. Steinbach, Simone Fulda, Oliver Bähr

**Affiliations:** 1 Dr. Senckenberg Institute of Neurooncology, University Hospital Frankfurt, Goethe University, Frankfurt, Germany; 2 Institute of Neurology (Edinger-Institute), University Hospital Frankfurt, Goethe University, Frankfurt, Germany; 3 Department of Radiation Therapy and Oncology, University Hospital Frankfurt, Goethe University, Frankfurt, Germany; 4 Experimental Neurosurgery, University Hospital Frankfurt, Goethe University, Frankfurt, Germany; 5 Biocenter, Martin Luther University Halle-Wittenberg, Halle, Germany; 6 Department of Neurology, University Hospital Zurich, Zurich, Switzerland; 7 Institute for Experimental Cancer Research in Pediatrics, Goethe-University, Frankfurt, Germany; Swedish Medical Center, United States of America

## Abstract

B10 is a glycosylated derivative of betulinic acid with promising activity against glioma cells. Lysosomal cell death pathways appear to be essential for its cytotoxicity. We investigated the influence of hypoxia, nutrient deprivation and current standard therapies on B10 cytotoxicity. The human glioma cell lines LN-308 and LNT-229 were exposed to B10 alone or together with irradiation, temozolomide, nutrient deprivation or hypoxia. Cell growth and viability were evaluated by crystal violet staining, clonogenicity assays, propidium iodide uptake and LDH release assays. Cell death was examined using an inhibitor of lysosomal acidification (bafilomycin A1), a cathepsin inhibitor (CA074-Me) and a short-hairpin RNA targeting cathepsin B. Hypoxia substantially enhanced B10-induced cell death. This effect was sensitive to bafilomycin A1 and thus dependent on hypoxia-induced lysosomal acidification. Cathepsin B appeared to mediate cell death because either the inhibitor CA074-Me or cathepsin B gene silencing rescued glioma cells from B10 toxicity under hypoxia. B10 is a novel antitumor agent with substantially enhanced cytotoxicity under hypoxia conferred by increased lysosomal cell death pathway activation. Given the importance of hypoxia for therapy resistance, malignant progression, and as a result of antiangiogenic therapies, B10 might be a promising strategy for hypoxic tumors like malignant glioma.

## Introduction

Malignant gliomas are among the most aggressive cancer types, often show resistance to therapy and have a dismal prognosis. Current treatment includes tumor resection, radiotherapy and chemotherapy with the DNA methylating drug temozolomide [1]. Furthermore, DNA alkylating nitrosoureas like lomustin and the VEGF-A antibody bevacizumab are commonly used [2], [3]. Beyond these compounds neither targeted therapy nor any other cytotoxic agent has shown compelling clinical activity yet. Therefore, agents with new mechanisms of action are urgently needed.

A wealth of natural compounds has shown substantial anticancer activity. Betulinic acid, a pentacyclic triterpenoid discovered in the bark of the white birch tree, has received particular attention because it shows multiple biological activities, including anticancer properties [4], [5]. Due to its low solubility in aqueous solvents and the poor understanding of its mode of action, the therapeutic applicability is, however, limited. Therefore, several semi-synthetic derivatives of betulinic acid have been generated [6]. B10 is a new glycosylated derivative of betulinic acid with enhanced cytotoxic activity that has recently been shown to trigger cell death via the lysosomal pathway [7]. As the study by Gonzalez and colleagues is the only work on B10 and limited to in vitro experiments, in vivo or even clinical experience on B10 is lacking.

Gonzalez and colleagues found that the morphology of B10-induced cell death is rarely typical for apoptosis. In comparison to TRAIL-induced cell death, B10 causes only a slight degree of DNA fragmentation. In addition, bcl-2 overexpression fails to prevent B10-induced cell death. Knockdown of caspase-3 results in a significant but incomplete decrease of B10 cytotoxicity. A transient increase of the acidic (lysosomal) compartment within 9 h of B10 treatment is followed by a decrease from 15 h onwards. Moreover, B10 treatment results in a shift of the lysosomal enzymes cathepsin Z and cathepsin B (CTSB) from lysosomes to cytoplasm and nucleus, further corroborating the hypothesis that B10 induces a permeabilization of lysosomes. These results are confirmed by the finding of a delay in B10-induced cell death under co-treatment with the cathepsin inhibitor CA074-Me, whereas other inhibitors of lysosomal enzymes do not impair B10 activity. Thus, lysosomal enzymes and their release contribute to B10-triggered cell death.

The major physiological inductor of autophagy is nutrient starvation [8]. Furthermore, it has been shown in vitro that hypoxia induces autophagy in cancer cells including two glioma cell lines [9], [10]. Hypoxia induces double-membraned autophagosomes as seen in electron microscopy, leading to GFP-LC3 punctate staining with LC3-II accumulation and an increased production of acidic vesicular organelles. Shortage of nutrients and oxygen is a central feature of malignant gliomas.

In this study, we investigated whether the sensitivity of glioma cells to B10 is altered under hypoxic conditions or nutrient deprivation. This question is of particular interest because the efficacy of current cytostatic drugs is often impaired under these conditions [11].

## Materials and Methods

### Reagents, cell lines and culture conditions

B10 was synthesized by BioService Halle (Halle, Germany). CA074-Me was purchased from Enzo Life Sciences (Lörrach, Germany). All reagents not specified were purchased from Sigma (Taufkirchen, Germany). LNT-229 cells have been described [12]; LNT-229 and LN-308 cells were a kind gift of Dr. N de Tribolet (Lausanne, Switzerland) [13]. Cell lines were maintained in Dulbecco's modified eagle medium (DMEM) containing 10% fetal calf serum (FCS) (Biochrom KG, Berlin, Germany), 100 IU/ml penicillin and 100 µg/ml streptomycin (Life Technologies, Karlsruhe, Germany) [12].

### Primary cell culture

R28 primary glioblastoma cells (passage 10) were a kind gift of Dr. Christoph Beier and have been described [14]. MNOF132 (passage 6) were derived from a glioblastoma and MNOF168 cells (passage 14) were derived from a gliosarcoma both resected at the Goethe University Hospital in Frankfurt. MNOF132 and MNOF168 cells were a kind gift of Dr. Stefan Momma. Primary cell cultures were maintained in DMEM-F12 medium containing 20 ng/ml of each human recombinant epidermal growth factor (EGF) and human recombinant basic fibroblast growth factor (bFGF) (both from ReliaTech, Wolfenbüttel, Germany) as well as 20% BIT Admixture 100 supplement (Pelo Biotech, Planegg/Martinsried, Germany). These culture conditions enable tumor cells to maintain most of the characteristics of the original tumor including differentiation, expression pattern, and genetic mutation profile [14], [15]. New/additional EGF and bFGF (each 20 ng/ml) was added to the cell culture twice per week. For experiments flasks/wells were coated with 5 µg/ml laminin (Sigma, Deisenhofen, Germany) for 3 h, primary cells were allowed to attach for two days before the beginning of any experiment. Hypoxia experiments were conducted in serum-free DMEM containing 2 mM glucose in accordance to the experimental setup with established glioma cell lines.

### Induction of hypoxia

Hypoxia was induced as previously described [12], [16]. Briefly, 0.1% O_2_ was induced by incubation in Gas-pak pouches for anaerobic culture (Becton-Dickinson, Heidelberg, Germany) [17]. Cell density measurement after 48 h cannot accurately be performed under 0.1% O_2_ due to cellular detachment. Therefore, moderate hypoxia (1% O_2_) used for cell density measurements was induced in a Labotect incubator (Goettingen, Germany). Cells were seeded and allowed to attach in DMEM containing 10% FCS for 24 h under normoxia. Subsequently, the medium was removed and the cells were incubated in serum-free DMEM adjusted to 2 mM glucose under normoxia, 1% O_2_ or 0.1% O_2_ for the indicated intervals. Equal cell densities were ensured by crystal violet (CV) staining when comparing LN-308 sub cell lines (Scrsh and CTSBsh) [12].

### Western Blot analysis

After the incubation period, cells were washed with ice-cold PBS and harvested into ice-cold PBS containing protease inhibitors. Lysates were prepared as described using lysis buffer P and subjected to SDS-PAGE analysis [17]. Membranes were probed with antibodies to HIF-1α (BD Transduction Laboratories # 610958) or actin (Santa Cruz Biotechnology, # sc-1616). The secondary anti-rabbit and anti-goat antibodies were purchased from Santa Cruz Biotechnology. Chemiluminescence solution was used for detection and was composed of 1 ml solution A (200 ml 0.1 M Tris-HCl pH 8.6, 50 mg Luminol), 100 µl solution B (11 mg p-hydroxycurmarinacid, 10 ml DMSO) and 0.3 µl H_2_O_2_ (30%).

### Irradiation

Irradiation was administered at room temperature with single doses of X-rays ranging from 2 to 6 Gy using a linear accelerator (SL 75/5, Elekta, Crawley, UK) with 6 MeV photons/100 cm focus–surface distance with a dose rate of 4.0 Gy/min [18]. Control cells were also brought to the irradiation chamber but were not placed under the X-ray beam.

### Cell density and cell viability assays

Cell density was assessed by CV staining as previously described [19], [20]. Cell viability measurement using propidium iodide (PI) uptake and flow cytometry has been described [12]. Analysis was performed by flow cytometry employing a BD Canto II flow cytometer and Summit 4.2. software. Cell viability analysis by lactate dehydrogenase (LDH) release assay was performed with the Cytotoxicity Detection Kit (LDH) (Roche, Mannheim, Germany) [11], [12]. Trypan blue solution (0.4%) was purchased from Sigma. For cell viability analysis, the culture medium was diluted by an equal volume of trypan blue solution and the cells were incubated for 10 min. After the incubation period, the cells were pelleted by centrifugation (300 g) and the supernatant was carefully replaced by PBS. Finally, the cells were photographed with a Keyence BZ 8000 fluorescence microscope (Lightfield Channel) using BZ observation application software and a 20× magnification. Viable and dead cells were determined by manual counting. For clonogenicity analysis, 500 cells were seeded per well of a 6 well plate. After incubation overnight, the cells were exposed to B10 or temozolomide or irradiation or combinations thereof. 24 h after treatment the medium was replaced by fresh DMEM containing 10% FCS. Experiments were stopped by CV staining when two clones came close to being indistinguishable. The ATP assay (CLS II kit) was purchased from Roche. For ATP measurement, immediately after normoxic or hypoxic incubation, the plates were placed on ice, the cells pelleted by centrifugation and lysed in ATP releasing reagent (Sigma). The ATP concentration was determined by a luciferase assay using MikroWin 2000 software and a Mithras luminometer [17].

### Lysotracker staining, microscopy and quantification

Lysotracker red was purchased from Invitrogen (Karlsruhe, Germany). Live cell staining was performed according to the manufacturer's protocol using 50 nM Lysotracker. In brief, for staining we added Lysotracker to the culture medium to a final concentration of 50 nM and cells were incubated at 37°C in the dark for 10 minutes. Afterwards cells were analysed with a Keyence BZ 8000 fluorescence microscope (Texas Red Channel) using BZ observation application software. Lightfield images of primary glioma cells were acquired using the corresponding channel of the microscope. Quantification of the Lysotracker signal of the acquired photographs (Texas Red Channel) was accomplished by measuring the mean pixel intensity values for six independent visual fields of equal cell density using ImageJ v 1.48r software (http://imagej.nih.gov/ij/).

### Cathepsin B activity assay

Cathepsin B activity was measured using the fluorogenic cathepsin B activity assay Kit (Calbiochem CBA001, Darmstadt, Germany). To ensure comparability between sub cell lines, samples were normalized for protein concentration. Protein concentration was measured using a Bradford protocol.

### Plasmids and lentivirus production

The pLKO.1 plasmid targeting cathepsin B (CTSB) was ordered from Sigma (TRCN0000003658), the pLKO.1 plasmid with a non-targeting shRNA sequence was from Addgene (# 1864). Lentiviral production was perfomed according to the Addgene protocol in HEK293 cells using the packaging plasmid pCMV-dR8.2 dvpr (Addgene #8455) and envelope plasmid pCMV-VSVG (Addgene #8454). Lentiviral infection was done in the presence of polybrene (Millipore, Darmstadt, Germany). For selection 2 µg/ml puromycin (Sigma) was added to the culture medium.

### RNA extraction and quantitative reverse transcription-PCR (qRT-PCR) analysis

qRT-PCR has been described [16]. Briefly, for RNA purification TRIzol and the RNAeasy Kit (Invitrogen, Karlsruhe, Germany) were used. cDNA was synthesized with the Vilo cDNA synthesis kit (Invitrogen) (10 min at 25°C followed by 2 h at 42°C). Subsequently, the reaction was stopped at 85°C for 10 min. qRT-PCR was performed in the IQ5 real-time PCR detection system (Biorad, Munich, Germany) using Absolute Blue Q-PCR Mastermix with SybrGreen+Fluorescein (Thermo Fisher Scientific, Hamburg, Germany) and the following primer pairs: CTSB: Fwd: 5′-ACAGCCCGACCTACAAACAG-3′, Rev: 5′-CCAGTAGGGTGTGCCATTCT-3′ (designed using Primer3 [21]); 18S: Fwd: 5′-CGGCTACCACATCCAAGGAA-3′, Rev: 5′-GCTGGAATTACCGCGGCT-3′; Succinate-dehydrogenase complex subunit A (SDHA): Fwd: 5′-TGGGAACAAGAGGGCATCTG-3′, Rev: 5′-CCACCACTGCATCAAATTCATG-3′, carboanhydrase IX (CAIX): Fwd: 5′-AAGAAGAGGGCTCCCTGAAG-3′, Rev: 5′-TAGCGCCAATGACTCTGGTC-3′; vascular endothelial growth factor (VEGF): Fwd: 5′-AGCCTTGCCTTGCTGCTCTA-3′, Rev:: 5′-GTGCTGGCCTTGGTGAGG-3′. 18S and SDHA were both used as housekeeping genes for normalization. Cycle threshold (Ct) values were normalized for amplification of the 18S ribosomal RNA and the data were analyzed using the Vandesompele method [22].

## Results

### B10 shows cytotoxic effects on glioma cells

After 48 h of exposure to B10 the concentrations resulting in a 50% decrease of cell density ranged from 7 to 21 µM (Fig. S1A). Moreover, B10 treatment resulted in a dramatic increase in PI uptake (Fig. S1B) and strong LDH-release (Fig. S1C). LN-308 and LNT-229 cells were selected for further studies. The threshold concentration of 10 µM B10 was chosen for further experiments (red rectangle in Fig. S1B, S1C).

### Hypoxia enhances B10 cytotoxicity

**Figure 1:**
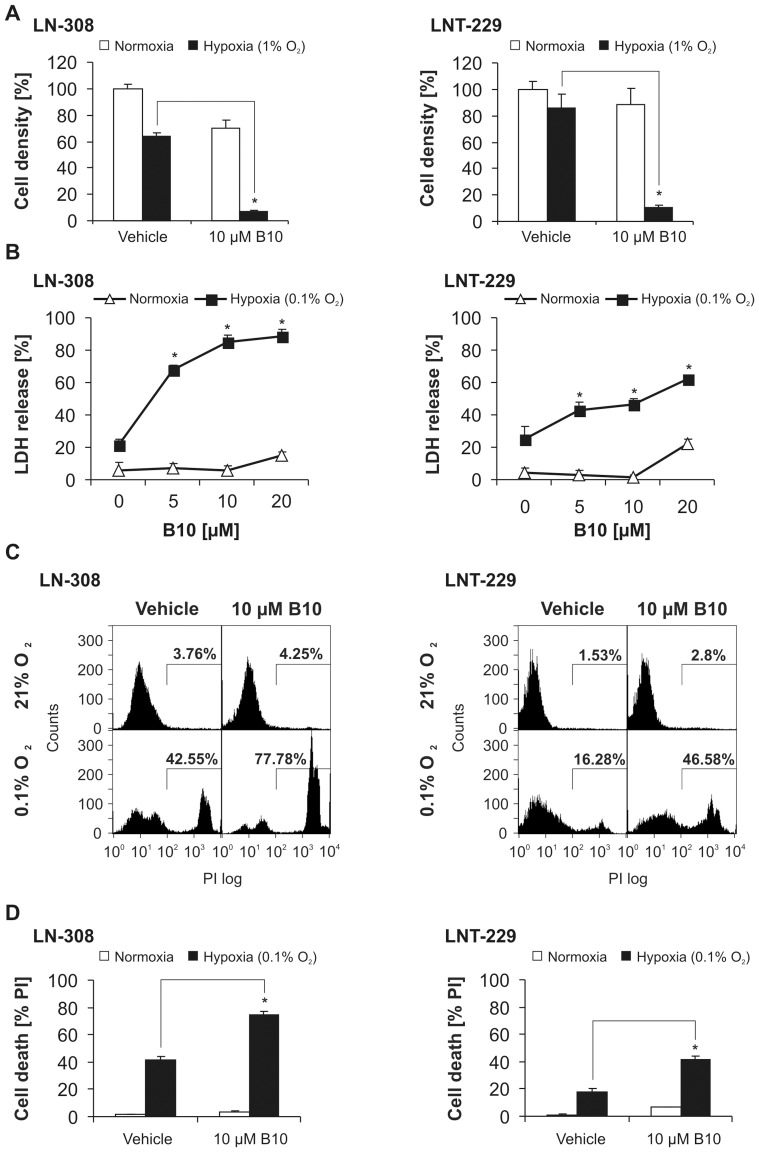
B10-induced cell death is substantially enhanced by hypoxia. A, LN-308 or LNT-229 cells were exposed to normoxia or 1% O_2_ with or without 10 µM B10 for 48 h. Cell density was measured by CV (LN-308 n = 3, LNT-229 n = 6, SD, *p<0.05). B, LN-308 or LNT-229 cells were treated with increasing concentrations of B10 under normoxia or 0.1% O_2_ for 20 h (LN-308) or 27 h (LNT-229). Cell death was quantified by LDH release (n = 4, SD, *p<0.05). C, LN-308 or LNT-229 cells were exposed to vehicle or 10 µM B10 under normoxia or 0.1% O_2_ for 23 h (LN-308) or 24 h (LNT-229). Cell death was measured by PI uptake. Representative flow cytometry profiles are shown. D, Quantification of cell death by PI uptake. LN-308 or LNT-229 cells were exposed to vehicle or 10 µM B10 under normoxia or 0.1% O_2_ for 23 h (LN-308) or 24 h (LNT-229) (LN-308 n = 3, SD; LNT-229 n = 4, SD, *p<0.05).

We exposed glioma cells to B10 either under normoxic or hypoxic conditions (1% or 0.1% O_2_) to investigate whether malignant glioma cells are particularly sensitive for B10 cytotoxicity under hypoxic conditions. Increased levels of HIF-1α as well as HIF-1α target gene induction under hypoxic conditions are shown in figure S2. CV staining showed a dramatic sensitization of LN-308 and LNT-229 cells to B10 under hypoxia with a decline in cell density (Fig. 1A). This sensitization also became evident by an increase in trypan blue staining (Fig. S3A). In addition, we found a strong and concentration-dependent increase of LDH release from LN-308 and LNT-229 cells after exposure to B10 under hypoxic conditions (Fig. 1B, Fig. S3B). These results were further corroborated by an increase of PI-positive cells in LN-308 and LNT-229 (Fig. 1C, D) as well as by an accelerated ATP depletion in hypoxia in the presence of B10 (Fig. S3C).

To further generalize these results, we treated primary brain cancer cells with B10 under normoxic or hypoxic conditions. The enhanced toxicity of B10 under hypoxic conditions also occurs in primary cell lines (Fig. S3D, E).

### Glucose deprivation does not alter B10 cytotoxicity

To address whether B10 cytotoxicity is influenced by nutrient depletion, we changed culture conditions by reducing glucose to 2 mM [11] which more accurately reflects in vivo tumor conditions. Glucose deprivation did not sensitize LN-308 cells to B10 toxicity as shown by cell density analysis (Fig. S4A) and PI uptake (Fig. S4B). Similar results were obtained by LDH release and for LNT-229 cells (data not shown).

### Combining B10 with temozolomide or irradiation shows additive cytotoxicity

To analyze any potential synergy of B10 and temozolomide, we investigated effects on clonogenic survival of LN-308 and LNT-229 cells and found additive inhibition of clonogenicity (Fig. S5A, B). Similarly, we tested for synergy of B10 and irradiation and again found additive effects for the reduction of clonogenicity (Fig. S5C, D). Further, we exposed LN-308 and LNT-229 cells to B10, temozolomide or their combination. CV and PI staining as well as LDH-release confirmed additive cytotoxicity (data not shown).

### Inhibition of lysosomal acidification rescues glioma cells from B10 toxicity under hypoxic conditions

**Figure 2:**
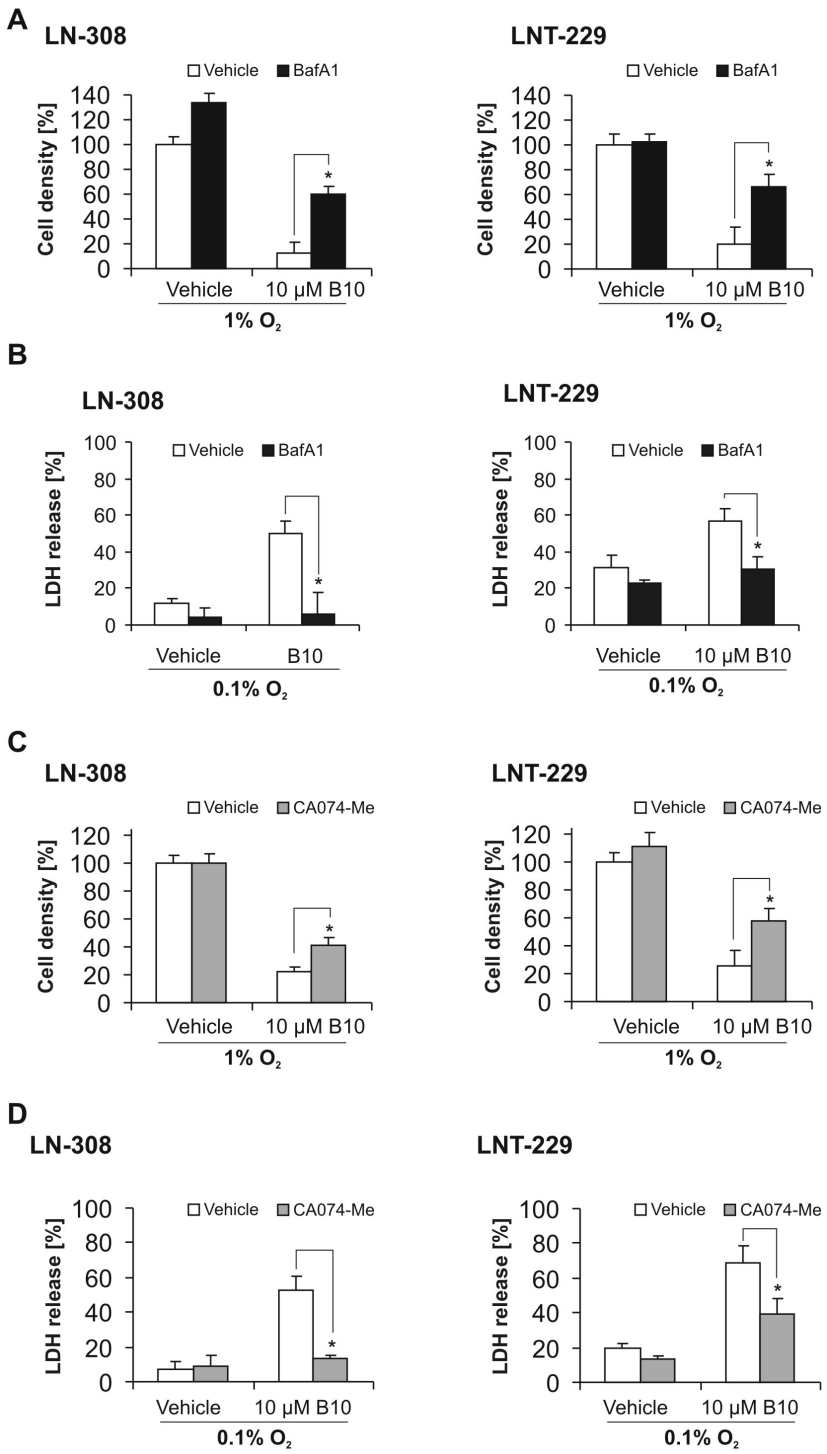
Blocking lysosomal acidification or inhibiting cathepsin B rescues glioma cells from B10 cytotoxicity under hypoxia. To block lysosomal acidification we used bafilomycin A1 (BafA1). A, LN-308 or LNT-229 cells were co-treated with 10 nM BafA1 and 10 µM B10 under hypoxic conditions (1% O_2_) for 48 h. Cell density was assessed by CV (n = 4, SD, *p<0.05). B, cell death was quantified by LDH-release after treating LN-308 or LNT-229 cells with BafA1 and B10 under hypoxic conditions (0.1% O_2_) for 19 h (LN-308) or 27 h (LNT-229) (n = 4, SD, * = p<0.05). To inhibit cathepsin B we used the cathepsin inhibitor CA074-Me. C, LN-308 or LNT-229 cells were co-treated with 10 µM CA074-Me and 10 µM B10 under hypoxic conditions (1% O_2_) for 48 h. Cell density was assessed by CV (n = 4, SD, *p<0.05). D, cell death was quantified by LDH release after treating LN-308 or LNT-229 cells with CA074-Me and B10 under hypoxic conditions (0.1% O_2_) for 19 h (LN-308) or 25 h (LNT-229) (n = 4, SD, *p<0.05).

To clarify the role of lysosomal cell death pathways for the increased cytotoxicity of B10 under hypoxic conditions, we examined whether glioma cells are rescued by inhibition of lysosomal acidification. BafA1 is an inhibitor of the vacuolar type H^+^-ATPase (V-ATPase) and thereby prevents acidification of lysosomes, resulting in reduced activity of lysosomal enzymes and blockade of the fusion of lysosomes and autophagosomes [23]. BafA1 almost completely rescued glioma cells from B10 cytotoxicity under hypoxic conditions. Cotreatment with B10 and BafA1 resulted in a cell density close to values of vehicle treatment (Fig. 2A). This was further corroborated by a reduction of LDH release (Fig. 2B) in LN-308 and LNT-229.

### Inhibition of cathepsins protects glioma cells against B10 cytotoxicity under hypoxic conditions

It has been shown that B10 ultimately leads to a permeabilization of lysosomes and release of cathepsins to the cytosol [7]. Therefore, we determined the effect of CA074-Me, an inhibitor of cathepsin B, L and S, on the cytotoxicity of B10 under hypoxic conditions. CA074-Me attenuated the effects of B10 on cell density and LDH-release under hypoxia in LN-308 and LNT-299 cells (Fig. 2C, D).

### Hypoxia increases lysosomal acidification and cathepsin B activity

**Figure 3:**
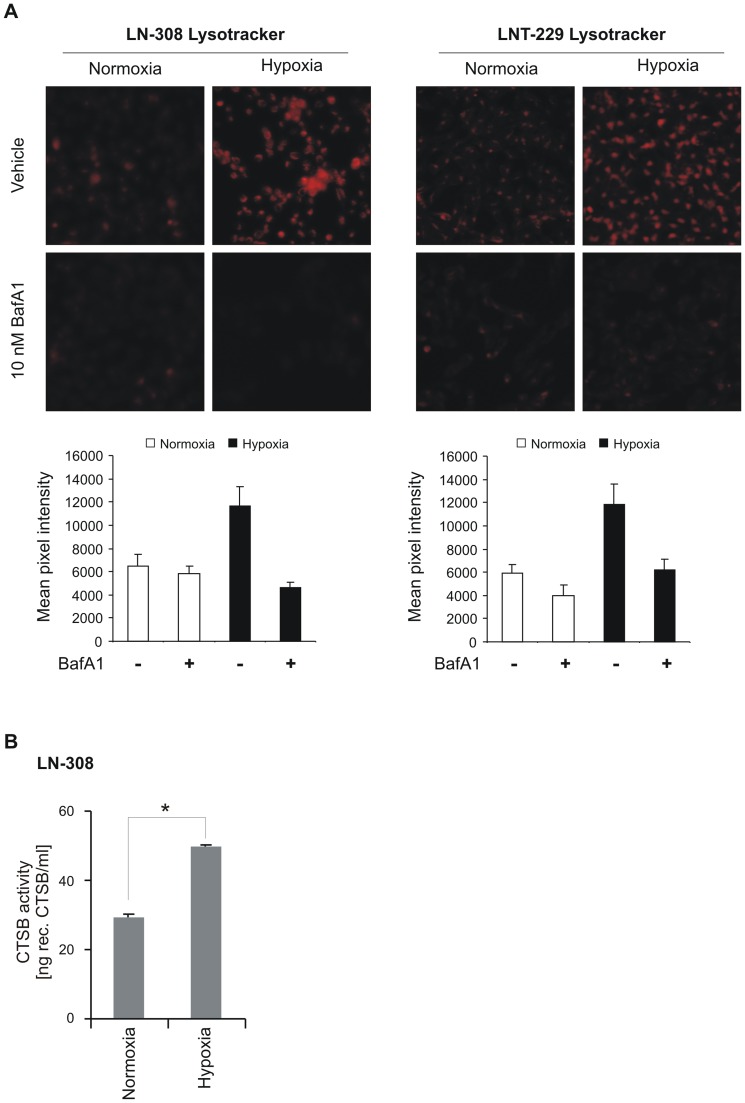
Hypoxia increases lysosomal acidification and cathepsin B activity. A, LN-308 or LNT-229 cells were grown under normoxia or hypoxia (0.1% O_2_) for 10 h and thereafter the lysosomal compartment was stained using Lysotracker red. Representative images are shown. Mean pixel intensities were measured with ImageJ software (n = 6, SD, *p<0.05). B, cathepsin B activity in LN-308 cells was measured after 8 h of hypoxia.

B10 cytotoxicity under hypoxic conditions seems to depend on lysosomal acidification. Therefore, we looked for changes of the lysosomal compartment and cathepsin B activity under hypoxia. Lysosomal acidification was increased under hypoxia as measured by Lysotracker staining in LN-308 and LNT-229 cells (Fig. 3A). As expected, BafA1 almost completely abolished Lysotracker staining (Fig. 3A). In addition, hypoxia also increased cathepsin B activity by more than 50% in LN-308 cells (Fig. 3B).

### Gene suppression of cathepsin B rescues glioma cells from B10 cytotoxicity under hypoxia

**Figure 4:**
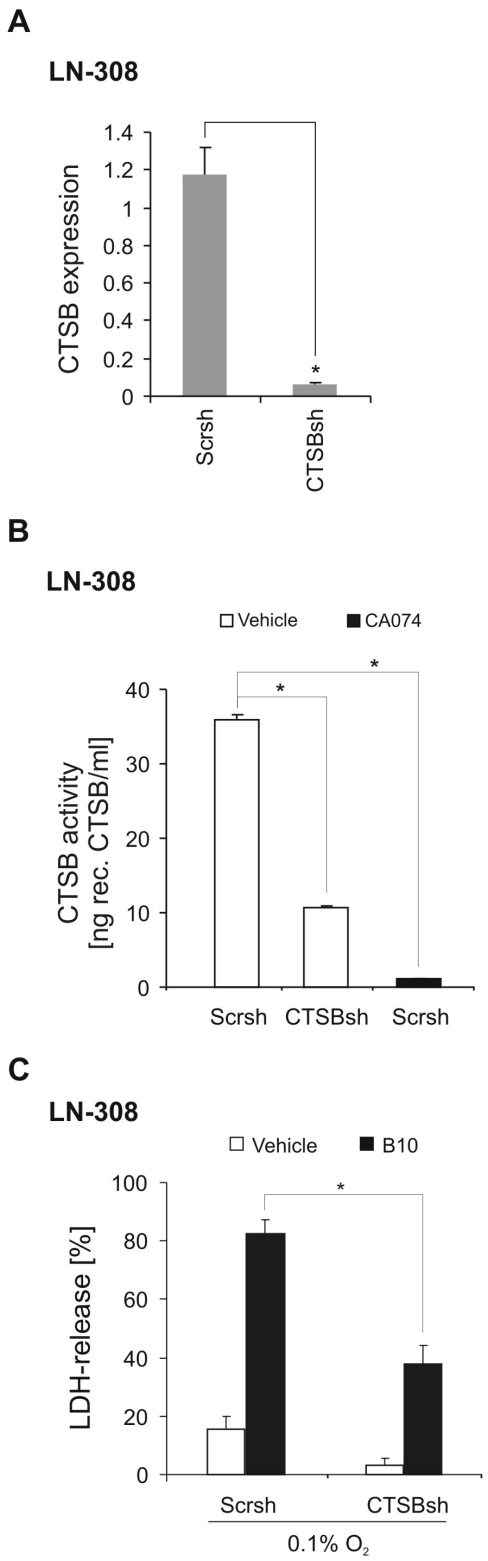
Gene suppression of cathepsin B rescues glioma cells from hypoxia sensitization to B10. Using a pLKO.1-based lentivirus LN-308 cathepsin B (CTSB) knockdown cells and control cells (non-targeting sequence, Scrsh) were generated. CTSB gene suppression was confirmed by qRT-PCR (A) and CTSB activity (B) (n = 3, SD, *p<0.05). The effect of the cathepsin inhibitor CA074-Me (10 µM) on cathepsin B activity in control cells (Scrsh) is shown in B. C, B10-mediated cytotoxicity under 0.1% O_2_ in LN-308 control cells and CTSBsh cells was quantified by LDH-release assay (n = 4, SD, *p<0.05).

To further examine the role of cathepsins in B10 cytotoxicity under hypoxic conditions we generated a stable knockdown of cathepsin B in LN-308. Efficacy of the knockdown sequence was tested by quantitative PCR (Fig. 4A). A cathepsin B activity assay proved the biological consequences of the knockdown (Fig. 4B).

Importantly, LN-308 CTSBsh cells displayed reduced LDH-release upon B10 exposure, corroborating the central role of cathepsin B in B10-induced cytotoxicity under hypoxic conditions (Fig. 4C). The fact that the effects in LN-308 CTSBsh cells do not equal those of CA074-Me (Fig. 2D) is most likely due to the incomplete inhibition of cathepsin B activity which was also reflected in the activity assay (Fig. 4B).

### REMBRANDT database analysis

**Figure 5:**
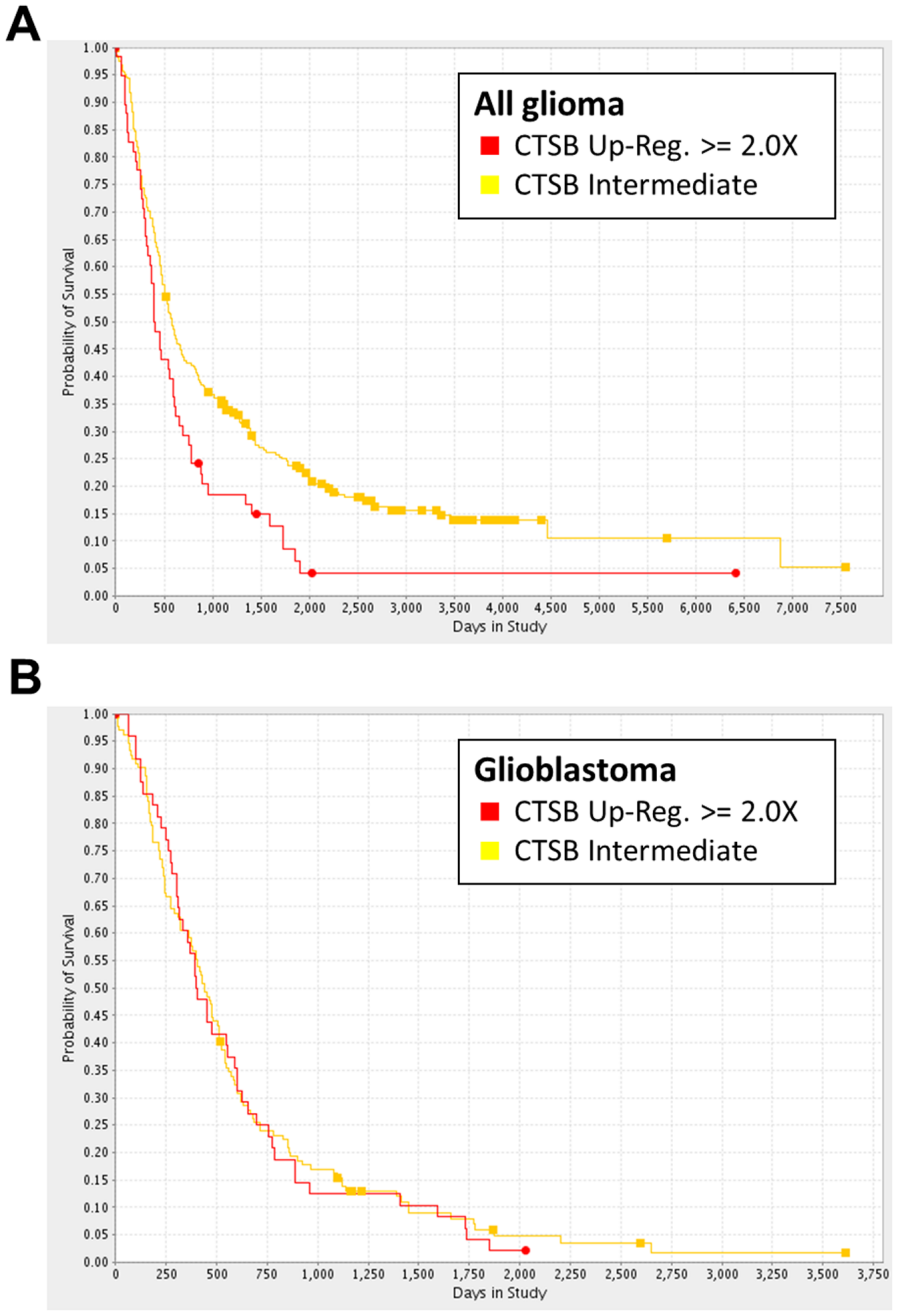
Survival analysis using the REMBRANDT database. A, The survival analysis in the group of all glioma patients reveals an inferior outcome for patients with a ≥ 2-fold upregulation of CTSB gene expression (CTSB-upregulated, n = 58; CTSB intermediate, n = 282; p = 0.004). B, No survival difference was found when restricting this analysis on glioblastoma patients (CTSB upregulated, n = 48; CTSB intermediate, n = 132). REMBRANDT database accessed June 18^th^ 2013.

An analysis using the REpository for Molecular BRAinNeoplasiaDaTa (REMBRANDT; https://caintegrator.nci.nih.gov/rembrandt/) shows that upregulated cathepsin B gene expression is associated with a shorter overall survival for the group of all glioma (Fig. 5A) [24]. This effect seemed to be caused by a high expression of cathepsin B in glioblastoma tissue. A survival analysis for cathepsin B gene expression in glioblastoma did not show a significant difference in overall survival in the REMBRANDT database on the basis of mRNA expression data (Fig. 5B).

## Discussion

**Figure 6:**
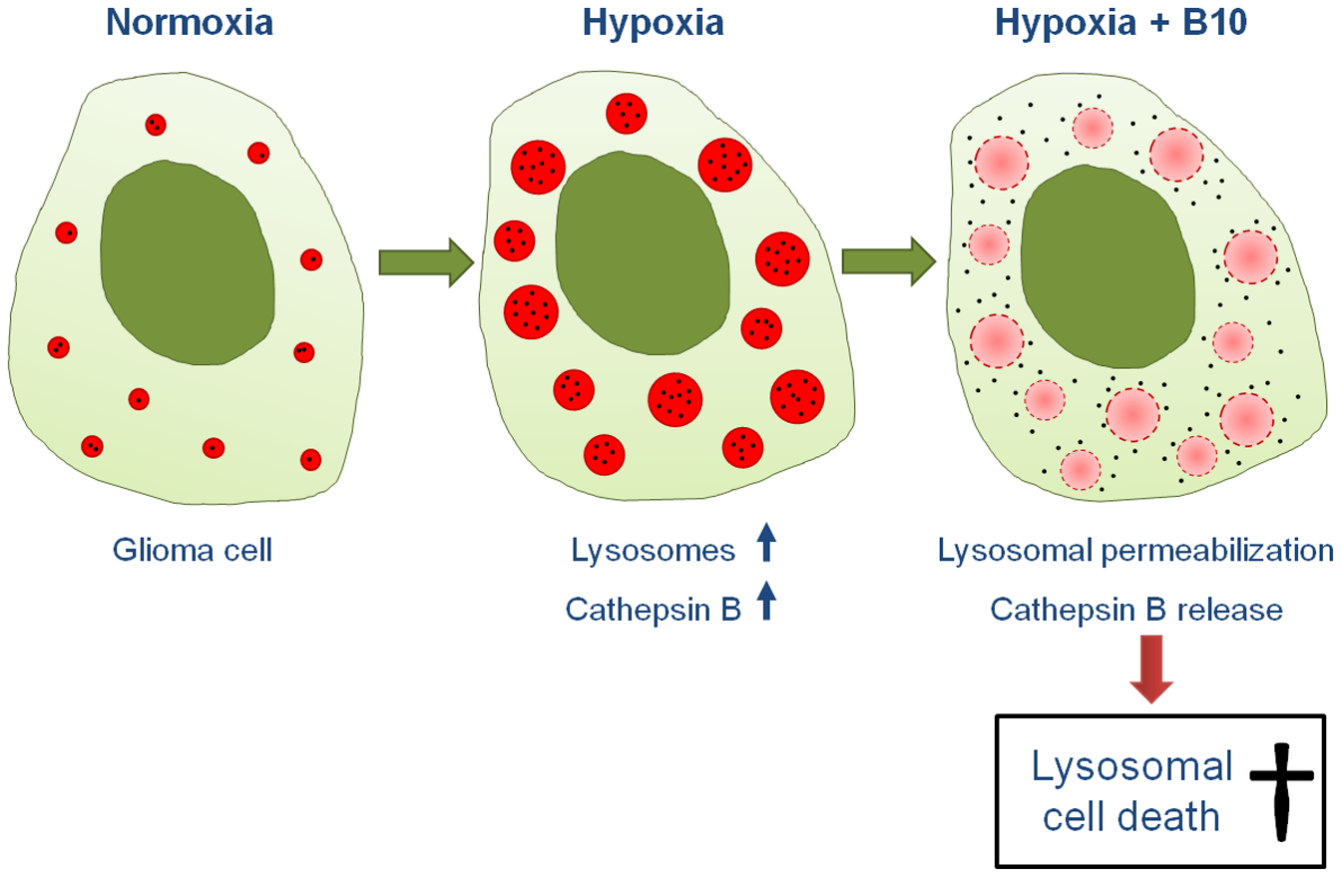
Proposed mode of action of B10 under hypoxic conditions. Under hypoxic conditions the lysosomal compartment and cathepsin B activity increase. This sensitizes glioma cells for B10, exploiting lysosomal permeabilization and cathepsin B release to induce lysosomal cell death.

Glioblastoma are among the most aggressive tumors and new therapeutic strategies are desperately needed. We here present new data on B10, a glycosylated derivative of the natural compound betulinic acid that was shown to have antiglioma activity in vitro by impairing lysosomal integrity [7]. We found that B10 cytotoxicity was augmented under hypoxic conditions. Furthermore, this effect was strongly dependent on lysosomal acidification as well as on cathepsin B expression as illustrated in a proposed model of action (Fig. 6). Despite the missing knowledge on a defined molecular target, B10 might therefore have a promising therapeutic potential due to its preference for hypoxic tumor cells that are usually difficult to treat.

One possible explanation why in vitro data often have limited validity in vivo is neglect for the actual conditions of the tumor microenvironment with nutrient deprivation and hypoxia. Both conditions are typical features of glioblastoma and are supposed to induce autophagy. No changes in B10 cytotoxicity were observed under low glucose conditions (Fig. S4). Intriguingly, we found a substantial increase of B10 cytotoxicity under hypoxia (Fig. 1). Lysosomal cell death pathways might be relevant for B10 cytotoxicity under hypoxia, since inhibition of lysosomal acidification or inhibition of cathepsins was capable to partially but distinctly rescue glioma cells (Fig. 2). Furthermore, hypoxia increased the lysosomal compartment and cathepsin B activity (Fig. 3). The inhibition of lysosomal acidification is an indirect way to inhibit cathepsins because a low pH is a prerequisite for their processing and full enzymatic activity. The relevance of our findings is underscored by the fact that cathepsin B activity is typically increased under hypoxia, which is a hallmark of glioblastoma [25], [26]. In addition, our analysis of the REMBRANDT database shows that upregulated cathepsin B gene expression is associated with a shorter overall survival for the group of all glioma (Fig. 5A). This effect seems to be caused by a high expression of cathepsin B in glioblastoma tissue. A survival analysis for cathepsin B gene expression in glioblastoma did not show a significant difference in overall survival at least in the REMBRANDT database on the basis of mRNA expression data (Fig. 5B). In contrast, however, Colin and colleagues showed that cathepsin B gene expression and cathepsin B protein levels are predictors for survival in two large independent cohorts [25]. In addition, they found that cathepsin B was highly expressed in tumor cells with an emphasis on the hypoxic pseudopalisading cells surrounding necrotic areas.

Glioblastomas usually show regions of hypoxia and current treatment standards often lose their activity in these areas due to reduced perfusion or unfavorable microenvironment. Moreover, antiangiogenic agents like bevacizumab seem to increase the extent and degree of hypoxia, thereby worsening this dilemma. Therefore, B10 seems to be a promising new agent because of its particular efficacy under hypoxic conditions.

## Supporting Information

Figure S1 **B10 is cytotoxic to human malignant glioma cells.** Glioma cells were exposed to increasing concentrations of B10 in standard culture conditions (10% FBS, 25 mM glucose, normoxia). Based on CV staining for cell density we calculated EC50 values for 10 glioma cell lines (A) (n = 3). Cell death was quantified by LDH-release in LNT-229 cells (B) (n = 4, SD) and PI uptake in LN-308 (C) (n = 3, SD).
(TIF)

Figure S2 **Hypoxia induces HIF1-α protein levels and transcriptional targets.** A, LN-308 and LNT-229 cells were exposed to normoxia or hypoxia (0.1% O_2_) for 8 h in serum-free DMEM containing 2 mM glucose. Cellular lysates were analyzed by immunoblot with antibodies for Hif1-α and actin. B, LN-308 or LNT-229 cells were exposed to normoxia, 1% or 0.1% O_2_ for 8 h in serum-free DMEM containing 2 mM glucose. Gene expression was analyzed by qRT-PCR with primers for CAIX or VEGF relative to 18S and SDHA. as housekeeping genes for normalization.
(TIF)

Figure S3 **B10-induced cell death is substantially enhanced by hypoxia in LN-308, LNT-229 and primary glioma cells.** A, LN-308 or LNT-229 cells were treated with vehicle or 10 µM B10 under normoxia or 1% O_2_ for 23 h (LN-308) or 38 h (LNT-229). Cell death was quantified by trypan blue staining (LN-308 n = 8, LNT-229 n = 6, SD, * = p<0.05). Representative photographs are included. B, LN-308 or LNT-229 cells were treated with vehicle or 10 µM B10 under normoxia or 0.1% O_2_ for 25 h. Cell death was quantified by LDH release (n = 8, SD, *p<0.05). C, LN-308 or LNT-229 cells were treated with vehicle or 10 µM B10 under normoxia or 0.1% O_2_ for 16 h. ATP was quantified by a luciferase-based assay (LN-308 n = 5, LNT-229 n = 7, SD, * = p<0.05). The ratio of ATP concentrations in B10-exposed cells to vehicle-treated cells is depicted. D, R28 and MNOF132 primary glioma cells were exposed to normoxia or 1% O_2_ with or without 10 µM B10 for 48 h. Cell density was measured by CV staining (n = 5, SD). Representative light microscopy photographs of R28 cells ultimately before CV staining are shown (right panel). E, R28 and MNOF168 cells were treated with 10 µM B10 under normoxia or 0.1% O_2_. LDH-release assay shows enhanced toxicity of B10 under hypoxia (n = 4, SD, *p<0.05).
(TIF)

Figure S4 **Glucose deprivation does not increase B10 cytotoxicity.** LN-308 cells were treated with 10 µM B10 in full medium for 3 days with different concentrations of glucose. Cell density was assessed by CV (A) (n = 3, ± SD) or by PI uptake (B) (n = 3, SD).
(TIF)

Figure S5 **Cotreatment of glioma cells with B10 with temozolomide or irradiation has additive anti-clonogenic effects.** A-D, LN-308 (A) and LNT-229 (B) cells were treated with increasing concentrations of temozolomide (TMZ) or increasing doses of irradiation (C, D) in the absence or presence of different concentrations of B10 for 24 h under normoxia. Thereafter, treatment medium was replaced by fresh DMEM containing 10% FCS. Experiments were stopped by CV staining when two clones came close to being indistinguishable (n = 3, SD).
(TIF)
