# Supplementary figures and images for: Hypoxia Enhances the Antiglioma Cytotoxicity of B10, a Glycosylated Derivative of Betulinic Acid
Source: PLoS One. 2014 Apr 17;9(4):e94921. doi: 10.1371/journal.pone.0094921 (PMC3990545; doi:10.1371/journal.pone.0094921)

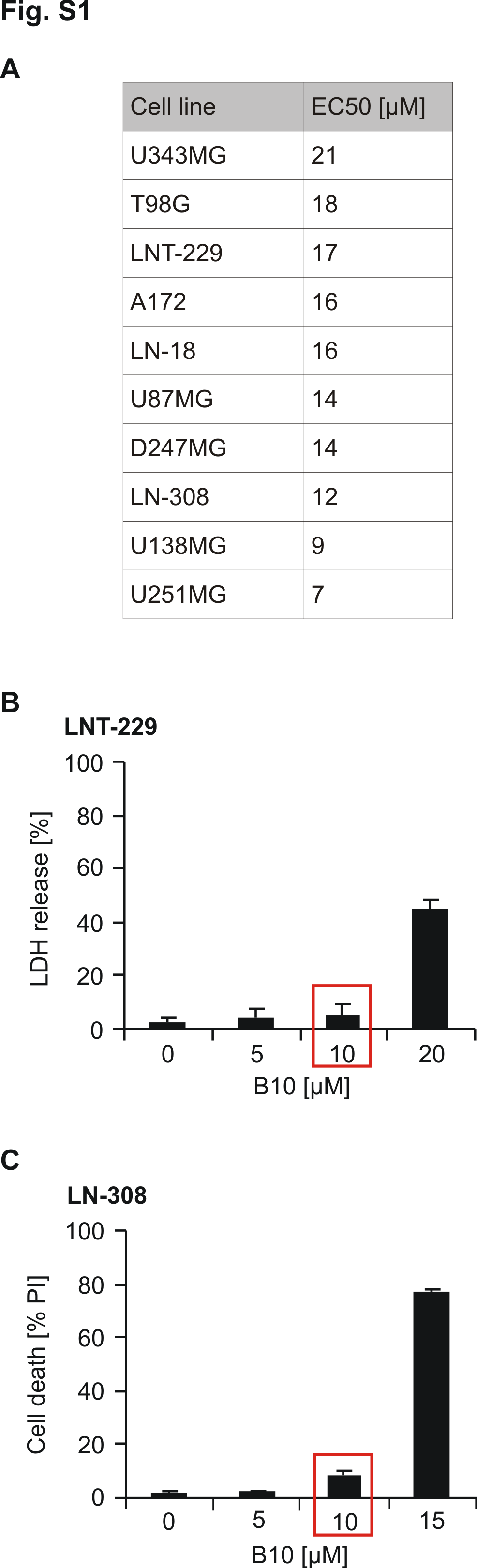

Supplement: Figure S1 — B10 is cytotoxic to human malignant glioma cells. Glioma cells were exposed to increasing concentrations of B10 in standard culture conditions (10% FBS, 25 mM glucose, normoxia). Based on CV staining for cell density we calculated EC50 values for 10 glioma cell lines (A) (n = 3). Cell death was quantified by LDH-release in LNT-229 cells (B) (n = 4, SD) and PI uptake in LN-308 (C) (n = 3, SD). (TIF) [file pone.0094921.s001.tif]

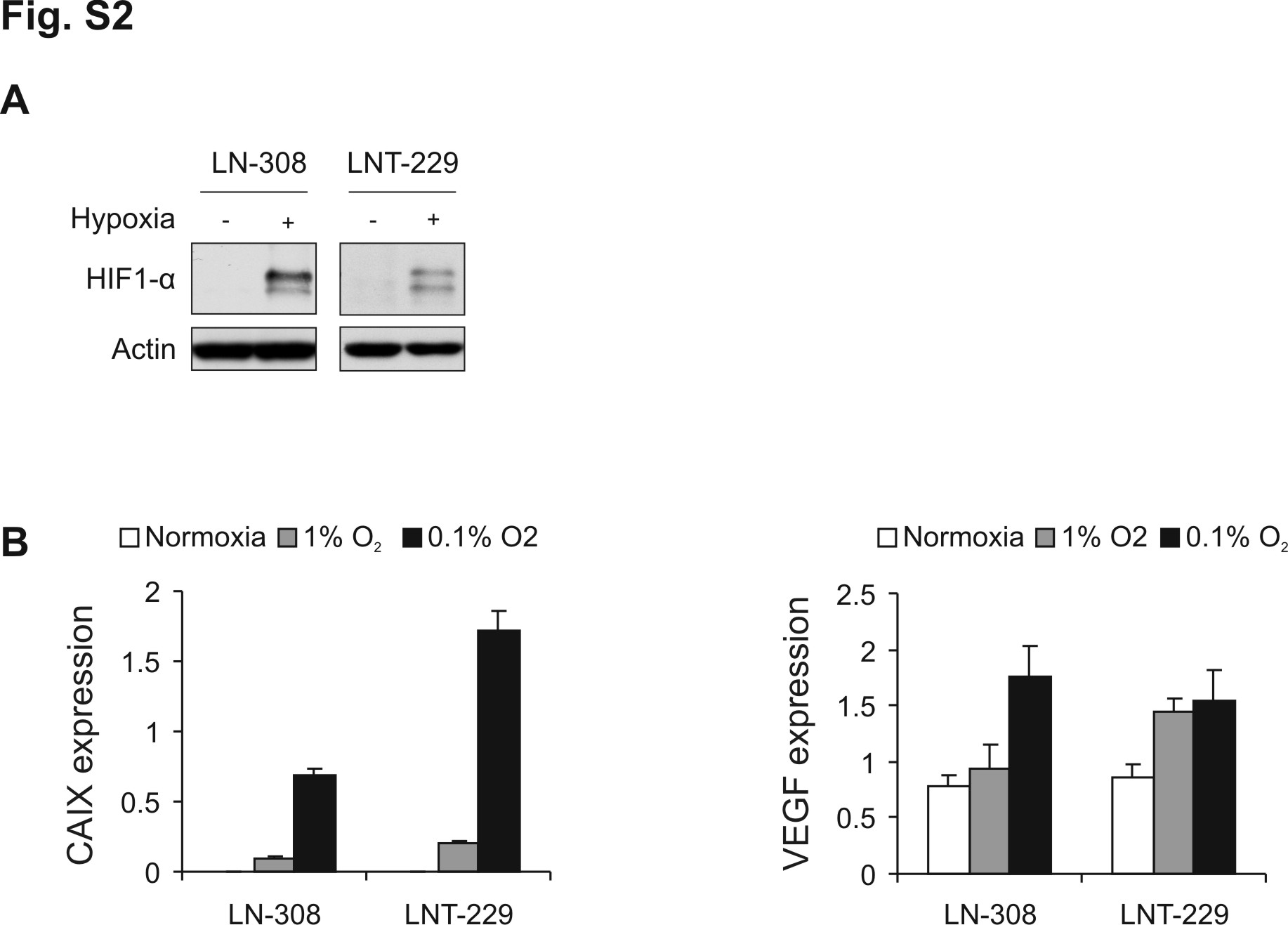

Supplement: Figure S2 — Hypoxia induces HIF1-α protein levels and transcriptional targets. A, LN-308 and LNT-229 cells were exposed to normoxia or hypoxia (0.1% O2) for 8 h in serum-free DMEM containing 2 mM glucose. Cellular lysates were analyzed by immunoblot with antibodies for Hif1-α and actin. B, LN-308 or LNT-229 cells were exposed to normoxia, 1% or 0.1% O2 for 8 h in serum-free DMEM containing 2 mM glucose. Gene expression was analyzed by qRT-PCR with primers for CAIX or VEGF relative to 18S and SDHA. as housekeeping genes for normalization. (TIF) [file pone.0094921.s002.tif]

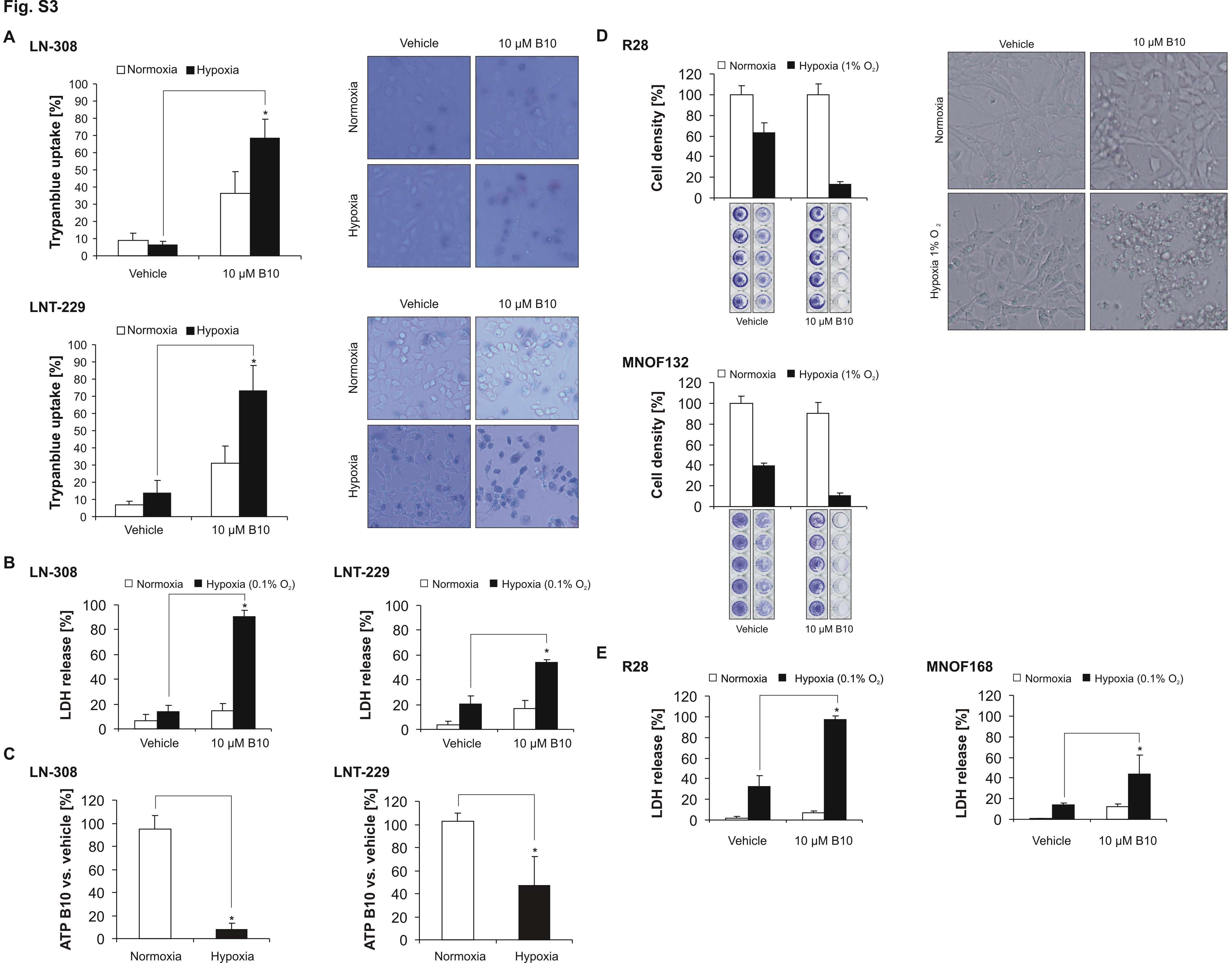

Supplement: Figure S3 — B10-induced cell death is substantially enhanced by hypoxia in LN-308, LNT-229 and primary glioma cells. A, LN-308 or LNT-229 cells were treated with vehicle or 10 µM B10 under normoxia or 1% O2 for 23 h (LN-308) or 38 h (LNT-229). Cell death was quantified by trypan blue staining (LN-308 n = 8, LNT-229 n = 6, SD, * = p<0.05). Representative photographs are included. B, LN-308 or LNT-229 cells were treated with vehicle or 10 µM B10 under normoxia or 0.1% O2 for 25 h. Cell death was quantified by LDH release (n = 8, SD, *p<0.05). C, LN-308 or LNT-229 cells were treated with vehicle or 10 µM B10 under normoxia or 0.1% O2 for 16 h. ATP was quantified by a luciferase-based assay (LN-308 n = 5, LNT-229 n = 7, SD, * = p<0.05). The ratio of ATP concentrations in B10-exposed cells to vehicle-treated cells is depicted. D, R28 and MNOF132 primary glioma cells were exposed to normoxia or 1% O2 with or without 10 µM B10 for 48 h. Cell density was measured by CV staining (n = 5, SD). Representative light microscopy photographs of R28 cells ultimately before CV staining are shown (right panel). E, R28 and MNOF168 cells were treated with 10 µM B10 under normoxia or 0.1% O2. LDH-release assay shows enhanced toxicity of B10 under hypoxia (n = 4, SD, *p<0.05). (TIF) [file pone.0094921.s003.tif]

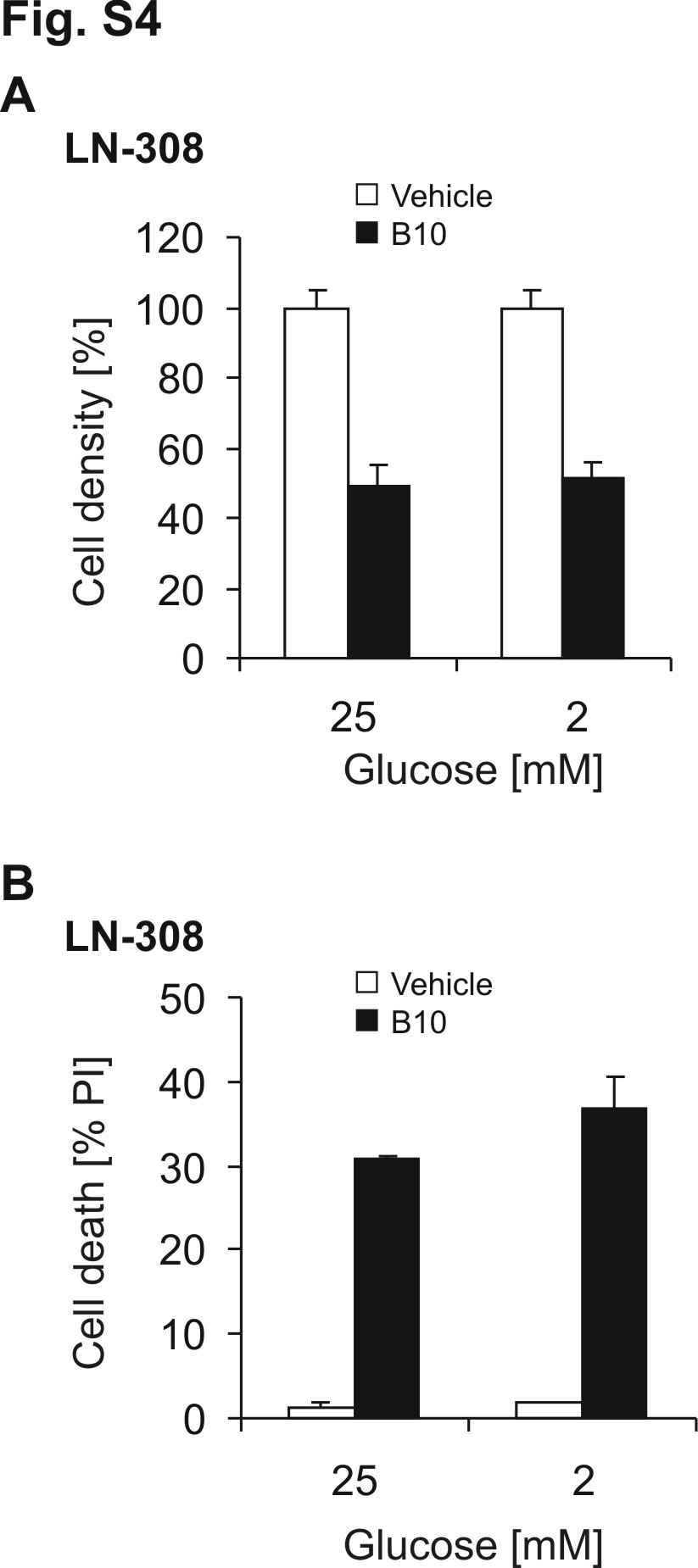

Supplement: Figure S4 — Glucose deprivation does not increase B10 cytotoxicity. LN-308 cells were treated with 10 µM B10 in full medium for 3 days with different concentrations of glucose. Cell density was assessed by CV (A) (n = 3, ± SD) or by PI uptake (B) (n = 3, SD). (TIF) [file pone.0094921.s004.tif]

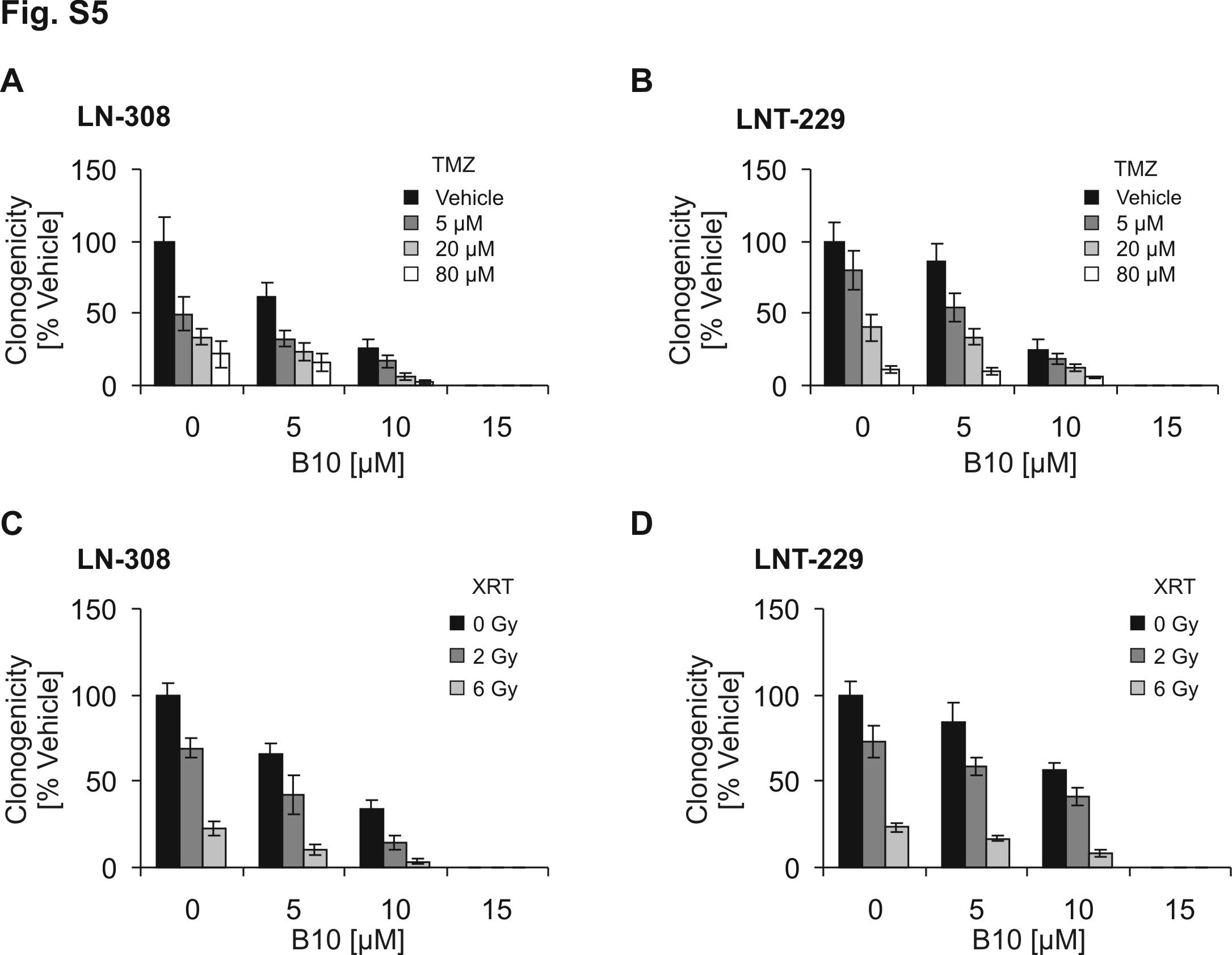

Supplement: Figure S5 — Cotreatment of glioma cells with B10 with temozolomide or irradiation has additive anti-clonogenic effects. A-D, LN-308 (A) and LNT-229 (B) cells were treated with increasing concentrations of temozolomide (TMZ) or increasing doses of irradiation (C, D) in the absence or presence of different concentrations of B10 for 24 h under normoxia. Thereafter, treatment medium was replaced by fresh DMEM containing 10% FCS. Experiments were stopped by CV staining when two clones came close to being indistinguishable (n = 3, SD). (TIF) [file pone.0094921.s005.tif]
